# Neuro-inspired continual anthropomorphic grasping

**DOI:** 10.1016/j.isci.2023.106735

**Published:** 2023-04-25

**Authors:** Wanyi Li, Wei Wei, Peng Wang

**Affiliations:** 1 State Key Laboratory of Multimodal Artificial Intelligence Systems, Institute of Automation, Chinese Academy of Sciences, Beijing 100190, China; 2 School of Artificial Intelligence, University of Chinese Academy of Sciences, Beijing 100049, China; 3 CAS Center for Excellence in Brain Science and Intelligence Technology, Chinese Academy of Sciences, Shanghai 200031, China; 4 Centre for Artificial Intelligence and Robotics, Hong Kong Institute of Science and Innovation, Chinese Academy of Sciences, Hong Kong 999077, China

**Keywords:** Neuroscience, Control engineering, Robotics

## Abstract

Humans can learn continuously grasping various objects dexterously. This ability is enabled partly by underlying neural mechanisms. Most current works of anthropomorphic robotic grasping learning lack the capability of continual learning (CL). They utilize large datasets to train grasp models and the trained models are difficult to improve incrementally. By incorporating several discovered neural mechanisms supporting CL, we propose a neuro-inspired continual anthropomorphic grasping (NICAG) approach. It consists of a CL framework of anthropomorphic grasping and a neuro-inspired CL algorithm. Compared with other methods, our NICAG approach achieves better CL capability with lower loss and forgetting, and gets higher grasping success rate. It indicates that our approach performs better on alleviating forgetting and preserving grasp knowledge. The proposed system offers an approach for endowing anthropomorphic robotic hands with the ability to learn grasping objects continually and has great potential to make a profound impact on robots in households and factories.

## Highlights

• Enabling anthropomorphic hand to learn grasping objects continually is critical
• A neuro-inspired continual anthropomorphic grasping (NICAG) approach is proposed
• NICAG performs better on alleviating forgetting and preserving grasp knowledge
• NICAG has a profound impact on robots in households and factories

## Graphical abstract

**Figure:**
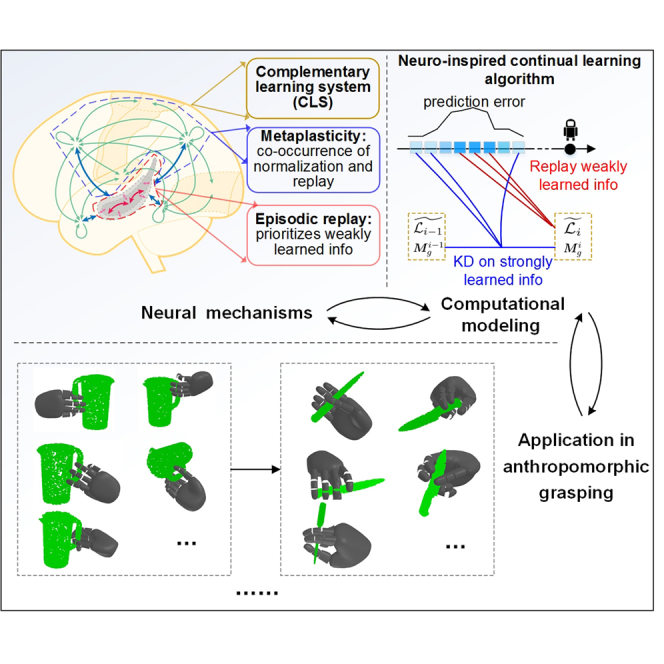

## Introduction

Humans are able to learn continuously grasping various objects dexterously throughout their lifetime under an endless variety of ever-changing scenarios. This ability is from two aspects. One is the exquisite flexibility and precision of the human hand. Another is the continual learning (CL) capability of the human brain, based on which, the dexterous grasping skill is acquired during childhood and further refined throughout life.

Biologists have tried to identify a number of underlying mechanisms that support CL. Some typical biological mechanisms include: complementary learning system (CLS),^1^ episodic replay,^2^ and meta-plasticity.^3^ CLS theory^1^ holds that two learning systems are possessed by mammalians, i.e., the hippocampus system and the neocortex system. The first allows for the rapid learning of the specifics of individual experiences which will, in turn, be played back over time to the second for acquiring structured knowledge gradually. Replay is the reactivation of neuronal activity patterns, in which neural patterns that had previously occurred during waking re-occur during later rest or sleep.^2^ Replay appears in the hippocampus and neocortical areas, is selective and partial, and benefits subsequent memory. Schapiro et al.^4^ suggest that human hippocampal replay during rest prioritizes weakly learned information. Metaplasticity is the ability of a synapse to be modified depending on its internal biochemical states,^3^ which then depends on the history of synaptic modifications and recent neural activity. An instantiation of metaplasticity is regularization or normalization, with which consolidated knowledge can be protected from forgetting through synapses with a cascade of states yielding different levels of plasticity.^5^ Especially, in biological networks, normalization and synaptic changes co-occur with replay.^6^

Anthropomorphic grasping is a critical skill for robotics because robots generally need to grasp an object in the majority of manipulation tasks.^7^^,^^8^ For a robot in an open and dynamical environment, it is necessary to learn new knowledge continually over time, as it is impossible to pre-program everything in advance. The capability to learn skills and knowledge over time without forgetting the previously learned is referred to as CL.^5^ Endowing an anthropomorphic hand with the ability to learn grasping continually could have an enormous societal impact. Examples include providing assistance in the household of disabled or elder people and resorting and packaging varied goods in factories.

Existing learning-based anthropomorphic robotic grasping approaches utilize supervised learning or reinforcement learning paradigms, and train the grasping policy with large amounts of annotated data. Grasp annotations of the training data are collected by humans,^9^ with simulation^10^ or physical robot tests.^11^ Given enough data, learning-based approaches achieved astonishing grasp ability. Nevertheless, they use large fixed-prepared dataset to train the grasp model and do not generalize well to novel objects. Furthermore, the trained grasping models are difficult to improve continually and incrementally over time.

Here, we proposed a neuro-inspired continual anthropomorphic grasping (NICAG) approach that integrates and adopts several discovered biological neural mechanisms supporting continual lifelong learning, i.e., CLS, episodic replay, and meta-plasticity. The proposed NICAG approach consists of a CL framework for anthropomorphic grasping and a neuro-inspired CL algorithm. Three layers, i.e., data layer, algorithm layer, and application layer, are included in the CL framework, thus making CL of anthropomorphic grasping possible. The neuro-inspired CL algorithm prevents forgetting and preserves grasp knowledge by replaying weakly learned information and knowledge distillation on strongly learned information, consequently, the anthropomorphic robotic hands can learn to grasp different objects continually and incrementally over long sequential grasp stream. We validate the proposed approach through dataset experiments and simulated experiments. Compared with other methods, our NICAG approach not only achieves better CL capability with lower average loss and forgetting but also gets a higher success rate (SR) for grasping. It indicates that our approach performs better on alleviating forgetting and preserving grasp knowledge. The proposed system offers an approach for endowing anthropomorphic robotic hands with the ability to learn to grasp different objects continually and incrementally over time and has great potential to make a profound impact on robots in households and factories. The contributions of this paper are as follows.

1. A NICAG approach incorporates several discovered biological mechanisms of lifelong learning.
2. A CL framework of anthropomorphic grasping that includes data layer, algorithm layer, and application layer. It makes CL of anthropomorphic grasping possible.
3. A neuro-inspired CL algorithm that prevents forgetting and preserves grasp knowledge by replaying weakly learned information and knowledge distillation on strongly learned information, thus the anthropomorphic robotic hands can learn to grasp different objects continually and incrementally over long sequential grasp stream.
4. A validation of the proposed NICAG approach through dataset experiments and simulated experiments, demonstrating our method achieves better performance than state-of-the-art CL methods. It not only achieves better CL capability with lower average loss and forgetting but also gets higher SR for grasping.

## Results

**Figure 1:**
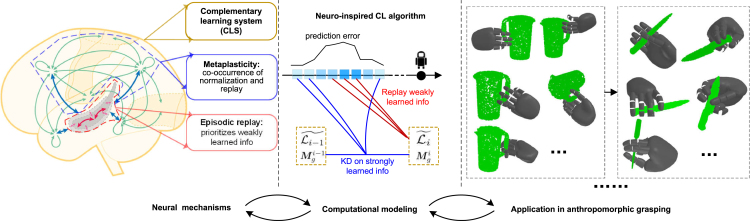
Schematic of the design process of neuro-inspired continual anthropomorphic grasping (NICAG) (Left) The mechanisms concerning lifelong learning, on which we focused. The picture for lateral view of one hemisphere of the brain is adapted from Kumaran et al.^12^ (Middle) Computational modeling incorporates focused mechanism into a neuro-inspired continual learning (CL) algorithm. The correspondence between specific neural mechanism and its computational component is indicated with same color, such as red is for episodic replay. (Right) Applying the designed CL algorithm into anthropomorphic grasping.

The three focused biological neural mechanisms supporting continual lifelong learning, i.e., CLS, episodic replay, and meta-plasticity, have been well described in ref.^1^^,^^2^^,^^3^^,^^4^^,^^6^ We propose our NICAG approach based on these mechanisms. The design process of our NICAG approach is shown in Figure 1. We will describe the CL framework of anthropomorphic grasping, the neuro-inspired CL algorithm and experimental results in the following.

### The problem and the continual learning framework of anthropomorphic grasping

**Figure 2:**
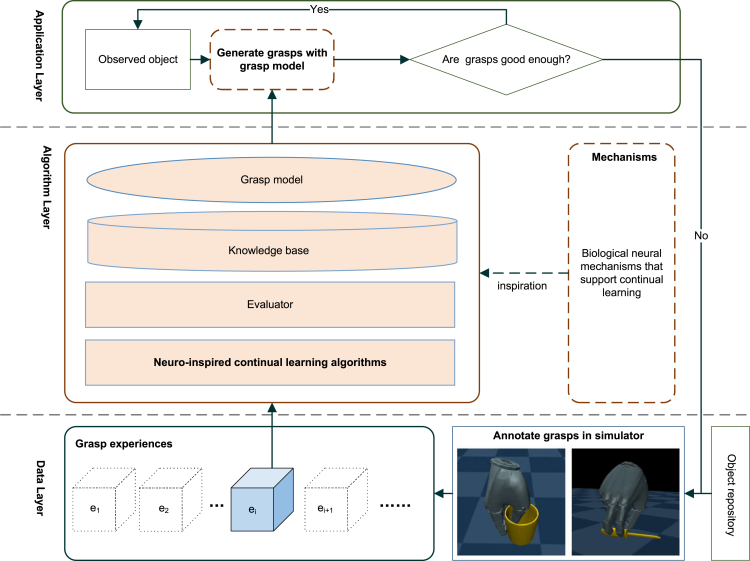
Continual learning framework of anthropomorphic grasping

To enable anthropomorphic robotic hands to learn to grasp objects continually over time, we design a neuro-inspired CL framework for anthropomorphic grasping (NICAG-framework), which is shown in Figure 2. There are three layers in the NICAG-framework: data layer, algorithm layer, and application layer. Data layer is responsible for generating the stream of anthropomorphic grasping experiences. Algorithm layer trains the grasp model based on information from data layer. Application layer applies trained grasp model to objects in the field, those objects with bad grasps are sent to the data layer for better learning.

Formally, in the NICAG-framework, a CL algorithm $A_{CL}$ is expected to update its internal state, e.g., its internal grasp model $M_{g}$ and knowledge base representing as specific data structures, based on a non-stationary sequentially accessible stream of anthropomorphic grasping experiences $E=\left(e_{1},e_{2}\ldots ,e_{i},\ldots ,e_{n}\right)$. The objective of $A_{CL}$ is to improve its performance on a set of grasp metrics $\left(p_{1},\ldots ,p_{m}\right)$ as evaluated on a test stream of experiences $\left(e_{1}^{t},\ldots ,e_{n}^{t}\right)$.

With respect to the stream of anthropomorphic grasping experiences $E=\left(e_{1},e_{2}\ldots ,e_{i},\ldots ,e_{n}\right)$, the i-th experience consists of $e_{i}=\left\{{\left\langle P_{k},g_{k}\right\rangle }_{k=1}^{n_{i}}\right\}$, where each pair constitutes a grasp example consists of a point cloud $P_{k}$ of the observed object, and a grasp $g_{k}$. The grasp is defined as g={p,θ}. The hand wrist pose p is given in special Euclidean group SE(3), consisting of the translation $t=\left[t_{x},t_{y},t_{z}\right]$ and orientation quaternion $q=\left[q_{w},q_{x},q_{y},q_{z}\right]$. The hand joint configuration θ is denoted by the actual degree of freedom of the anthropomorphic robot hand. In this work we use anthropomorphic robot hand DLR/HIT Hand II, of which $\theta \in R^{20}$.

Within the learning framework, this paper proposes a neuro-inspired CL algorithm $A_{CL}$. It is used to update internal grasp model $M_{g}$ and knowledge base. The grasp model $M_{g}$ takes point cloud P of the observed object as input and predicts high quality grasps. The knowledge base is represented as a memory buffer M and a last trained model. The details of the neuro-inspired CL algorithm $A_{CL}$ are provided in the next subsection.

### The neuro-inspired continual learning algorithm

**Figure 3:**
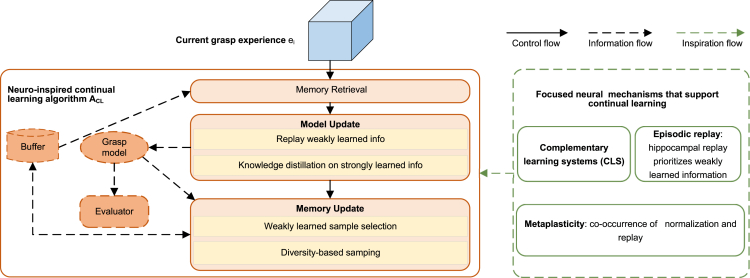
Schematic view of the proposed method

We first describe our neuro-inspired CL algorithm $A_{CL}$ at a high level here. $A_{CL}$ prevents forgetting and preserves grasp knowledge in both sample-space and function-space. Replaying weakly learned information is for preserving grasp knowledge in sample space, while knowledge distillation on strongly learned information is for keeping knowledge in function-space. The schematic view of the proposed algorithm is given in Figure 3. It consists of three major steps: memory retrieval based on learnability criterion, model update by replay weakly learned information and knowledge distillation on strongly learned information, and memory update with weakly learned sample selection and diversity-based sampling. In the following subsections, we first introduce the learnability criterion, and then provide details of three major steps.

#### Learnability criterion

To indicate whether a training sample is strongly learned or weakly learned, we adopt the learnability criterion from Sun et al.,^13^ which measures how much the grasp model $M_{g}$ can explain the training sample $\left\langle P_{⋆},g_{⋆}\right\rangle$ once it has absorbed its information in the memory. Adapted from Sun et al.,^13^ we define the learnability as follows:

(Equation 1) $$s_{learn}\left(\left\langle P_{⋆},g_{⋆}\right\rangle ;M\right)=\log\;p\left(g_{⋆}^{\prime }|g_{⋆},g_{M};P_{⋆},P_{M}\right){|}_{g_{⋆}^{\prime }=g_{⋆}}$$

where, $g_{⋆}^{\prime }$ and $g_{⋆}$ are used to represent two realizations of the same random variable describing a grasp.

Due to the used grasp model is a variational autoencoder (VAE) based generative model, after the grasp model $M_{g}$ have visited a grasp experience, the learnability of sample $\left\langle P_{⋆},g_{⋆}\right\rangle$ with respect to $M_{g}$ is calculated specifically as a quantity related with its loss:

(Equation 2) $$s_{learn}\left(\left\langle P_{⋆},g_{⋆}\right\rangle ,M_{g}\right)=e^{-L\left(\left\langle P_{⋆},g_{⋆}\right\rangle ,M_{g}\right)}$$

where $L\left(\left\langle P_{⋆},g_{⋆}\right\rangle ,M_{g}\right)$ is the loss of sample $\left\langle P_{⋆},g_{⋆}\right\rangle$ after passing through the grasp model $M_{g}$. The information of memory buffer M in Equation 1 has been incorporated into the grasp model $M_{g}$ during the training process on the latest visited grasp experience.

#### Memory retrieval based on learnability criterion

Based on learnability score, there are two parts in memory buffer M: weakly learned samples $S_{wl}$ and diverse strongly learned samples $S_{sl}$. They are with same size $\frac{\left|M\right|}{2}$, where |M| is the size of memory buffer M. The memory buffer is updated once an experience was learned, details are in Section "memory update with weakly learned sample selection and diversity-based sampling". For each incoming mini-batch $B_{j}$ drawn from current grasp experience $e_{i}$, the memory retrieval step randomly selects $\frac{\left|B_{j}\right|}{2}$ weakly learned samples (denoted as $B_{j}^{wl}$) and $\frac{\left|B_{j}\right|}{2}$ diverse strongly learned samples (denoted as $B_{j}^{sl}$ ) from the memory buffer M, $\left|B_{j}\right|$ is the batch size. To enhance the diversity of retrieved samples, 3D data augmentation on $B_{j}^{wl}$ and $B_{j}^{sl}$ are applied. Operations for 3D data augmentation include jitter, dropout and rotation. Jitter operation adds a clipped Gaussian noise with zero mean and standard deviation σ to the position of each point. Dropout augmentation throw away points randomly with max ratio $r_{\max}$. And rotation augmentation randomly rotates the object $P_{k}$ and grasp $g_{k}$ along three axes. The retrieved and augmented samples are used for updating grasp model $M_{g}$, as described in Section "model update by replay weakly learned information and knowledge distillation on strongly learned information".

#### Model update by replay weakly learned information and knowledge distillation on strongly learned information

To preserve grasp knowledge in sample space, we replay the retrieved weakly learned samples $B_{j}^{wl}$, the loss of weakly learned information replay is defined as:

(Equation 3) $$L_{replay}=L\left(B_{j}^{wl},M_{g}\right)$$

To keep grasp knowledge in function-space, we apply knowledge distillation on strongly learned samples $B_{j}^{sl}$. With respect to the strongly learned samples, it is expected that the current grasp model $M_{g}^{i}$ and the previous grasp model $M_{g}^{i-1}$ encodes latent code and generate final grasp in the same way. We utilize a KL-divergence loss to enforce the latent code distributions of $M_{g}^{i}$ and $M_{g}^{i-1}$ to be close, and use a reconstruction loss to encourage the output of $M_{g}^{i}$ and $M_{g}^{i-1}$ to be same. The KL-divergence loss and the reconstruction loss are formulated in Equations 4 and 5. The loss of knowledge distillation consist of two terms, i.e., the KL-divergence loss and the reconstruction loss, and is defined in Equation 6.

(Equation 4) $$L_{kl}=KL\left(Q\left(E_{i},\left(B_{j}^{sl}\right)\right)∥P\left(E_{i-1},\left(B_{j}^{sl}\right)\right)\right)$$

where $E_{i}$ and $E_{i-1}$ are the encoders of current grasp model $M_{g}^{i}$ and the previous grasp model $M_{g}^{i-1}$, respectively, $KL\left(Q∥P\right)$ is the Kullback-Leibler (KL) divergence to measure how different these two distributions *Q* and *P* are.

(Equation 5) $$L_{r}=\lambda_{v}\frac{1}{N}\sum_{k=1}^{N}∥M_{g}^{i}{\left(B_{j}^{sl}\right)}_{k}^{V}-M_{g}^{i-1}{\left(B_{j}^{sl}\right)}_{k}^{V}∥+\lambda_{\theta }\cdot {\left|M_{g}^{i}{\left(B_{j}^{sl}\right)}^{\theta }-M_{g}^{i-1}{\left(B_{j}^{sl}\right)}^{\theta }\right|}_{1}$$

The reconstruction loss is based on the reconstructed hand mesh. It consists of two terms: hand mesh vertices displacement and joint angles error. In Equation 5, $M_{g}^{i}{\left(B_{j}^{sl}\right)}^{V}$ is the vertices set of the reconstructed hand mesh, and $M_{g}^{i}{\left(B_{j}^{sl}\right)}^{\theta }$ is the joint angle of generated grasps.

(Equation 6) $$L_{kd}=L_{kd}\left(B_{j}^{sl},M_{g}^{i-1},M_{g}^{i}\right)=\lambda_{kl}\cdot L_{kl}+L_{r}$$

where $M_{g}^{i-1}$ is the previous version of grasp model, parameters of which just were updated based on the last experience $e_{i-1}$, $M_{g}^{i}$ is the current version of grasp model which is visiting the current experience $e_{i}$. $\lambda_{kl}$ of Equation 6, $\lambda_{v}$ and $\lambda_{\theta }$ of Equation 5 are constants to balance the losses.

Combining with the loss of mini-batch $B_{j}$ drawn from current grasp experience $e_{i}$, loss of weakly learned replay, and loss of knowledge distillation on strongly learned information, we perform model update by optimizing the following loss with respect to the parameters of the grasp model $M_{g}$:

(Equation 7) $$L_{grasp}^{cl}=L\left(B_{j},M_{g}\right)+\lambda_{replay}\cdot L_{replay}+L_{kd}$$

#### Memory update with weakly learned sample selection and diversity-based sampling

After the grasp model $M_{g}$ has been trained on a grasp experience, we perform the memory update step. Firstly, we merge the samples from memory buffer M and samples from the current experience $e_{i}$ as $S_{M}\cup S_{e_{i}}$ and calculate their learnability scores according to Equation 1. Secondly, from $S_{M}\cup S_{e_{i}}$, we select top $\frac{\left|M\right|}{2}$ samples with lowest learnability score as weakly learned samples, noted as $S_{wl}$. And then, we select $\frac{\left|M\right|}{2}$ examples from rested samples $S_{rest}=S_{M}\cup S_{e_{i}}\text{∖}S_{wl}$ descending by learnability score with an interval of $\left|S_{rest}\right|/\left(\frac{\left|M\right|}{2}\right)$, as a result, $\frac{\left|M\right|}{2}$ diverse strongly learned samples are included, which is denoted as $S_{sl}$. Finally, the contents of memory buffer is replaced with the selected |M| samples, i.e., $S_{wl}$ and $S_{sl}$.

### Experimental results

A validation of the proposed NICAG approach through dataset experiments and simulated experiments, demonstrating our method achieves better performance than state-of-the-art CL methods. It not only achieves better CL capability with lower average loss and forgetting but also gets higher SR for grasping.

To validate our proposed NICAG approach, we conduct experiments on both dataset and in simulation against a set of CL methods. The experiments aim to evaluate the CL capability and the grasp performance of the NICAG approach.

For evaluation metrics in dataset experiments, Average Loss (L_i) and Average Forgetting (F) are used to evaluate the CL capability of compared methods. The Average Loss (L_i) is the averaged loss of grasp model on test sets of learned experiences $\left(e_{1},e_{2}\ldots ,e_{i-1}\right)$ so far after the completion of CL at experience $e_{i}$. The Mean Average Loss (mAL) is mean of Average Loss over all experiences $\left(e_{1},e_{2}\ldots ,e_{n}\right)$ that is defined in Equation 8. The Average Forgetting (F) is defined upon Average Loss in Equation 9.

(Equation 8) $$mAL=\sum_{i=1}^{n}L_{i}$$

(Equation 9) $$F=\frac{1}{n-1}\sum_{i=2}^{n}\min\left(L_{i}-L_{i-1},0\right)$$

We use three quantitative metrics for evaluation in simulation: Success Rate (SR), Penetration Depth and Penetration Volume between the hand mesh and the target object. The three used metrics keep consistent with previous literature Mousavian et al.^14^ and Hasson et al.^15^ SR is commonly used in grasping tasks to measure the stability and quality of the generated grasps. For penetration depth and penetration volume, the implementation of Jiang et al.^16^ is used. When the hand collides with the target object, the penetration depth is computed as the maximum of the distances from vertices of hand mesh to the object surface.

We describe the implementation details of all compared CL algorithms here. All CL algorithms are implemented using Avalanche,^17^ which is an end-to-end CL library based on PyTorch. For IId-Offline, i.e., the variational grasp generator in DVGG^10^ (deep variational grasp generation), we use the implementation of Wei et al.^10^ For training of the compared methods, 150 epochs is used, and learning rate is set to 0.002 at start and divided by 10 when the validation error plateaus. Batch size is 512. We train all models on an RTX-3090 GPU. We present the detailed hyperparameters in Table 1.

**Table 1:** Hyperparameters for the compared methods

Methods	Hyperparameter grid	Tuned hyperparameter
Fine-tune	—a	—
IID-Offline	—	—
EWC	λ:[0.1,1,100]	λ=0.1
SI	c:[0.1,0.5,1]	c=0.5
ER	$\lambda_{replay}:\left[0.1,0.5,1\right]$	λ_replay=0.5
ER-RM	$\lambda_{replay}:\left[0.1,0.5,1\right]$	$\lambda_{replay}=0.5$
NI-WL^b^	$\lambda_{replay}:\left[0.1,0.5,1\right]$	$\lambda_{replay}=0.5$
NI-WL-RD^b^	$\lambda_{replay}:\left[0.1,0.5,1\right]$	$\lambda_{replay}=0.5$
NI-WL-RM^b^	$\lambda_{replay}:\left[0.1,0.5,1\right]$	$\lambda_{replay}=0.5$
NI-WL-RM-KD^b^	$\lambda_{replay}:\left[0.1,0.5,1\right],\lambda_{kl},\lambda_{\theta }:\left[0.1,0.5,1\right]\text{,}$λ_v:[1,10,30]	$\lambda_{replay}=0.5,\lambda_{kl}=0.1,\lambda_{\theta }=0.5,\lambda_{v}=30$

a “—” means not applicable.

b NI-WL, NI-WL-RD, NI-WL-RM, and NI-WL-RM-KD are four variants of our proposed method.

#### Results on dataset

To evaluate the CL capability of the proposed neuro-inspired algorithm, we compare it with other six methods on dataset. Details of the dataset are described in Section "dataset" of STAR Methods. The compared methods include four typical CL methods, namely, elastic weight consolidation (EWC),^18^ synaptic intelligence (SI),^19^ experience replay (ER),^20^ rainbow memory (ER-RM),^21^ and two baselines, i.e., Fine-tune and IId-Offline. Our proposed neuro-inspired CL algorithm includes four variants: NI-WL is the weakly learned replay, NI-WL-RD is the integration of weakly learned replay and randomly selective ER, NI-WL-RM is the integration of weakly learned replay and rainbow memory replay, NI-WL-RM-KD is the integration of weakly learned replay and knowledge distillation on strongly learned information. The description of compared methods is in Section "description of compared methods" of STAR Methods. We will report and analyze the evolution of test loss and forgetting along with training, the mAL and average forgetting, and the loss on the combined test set when the grasp model is finally trained on all grasp experiences, respectively.

**Figure 10:**
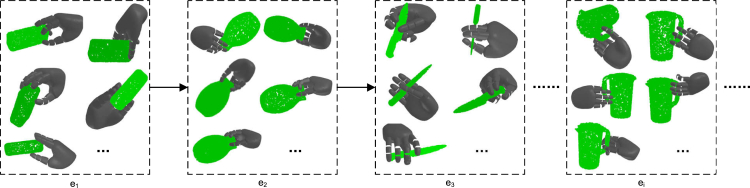
Grasp samples of each object in dataset as an experience

##### Evolution of test loss and forgetting along with training

**Figure 4:**
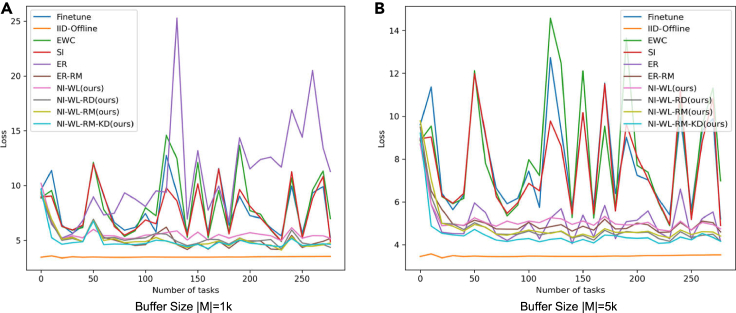
The average loss on test set without random rotation so far measured by the end of each task (object) (A) 1K buffer size is used for replay related methods. (B) 5K buffer size is used for replay related methods.

**Figure 5:**
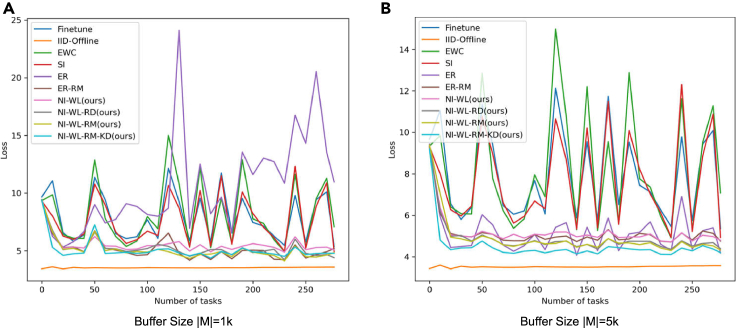
The average loss on test set with random rotation so far measured by the end of each task (object) (A) 1K buffer size is used for replay related methods. (B) 5K buffer size is used for replay related methods.

**Figure 6:**
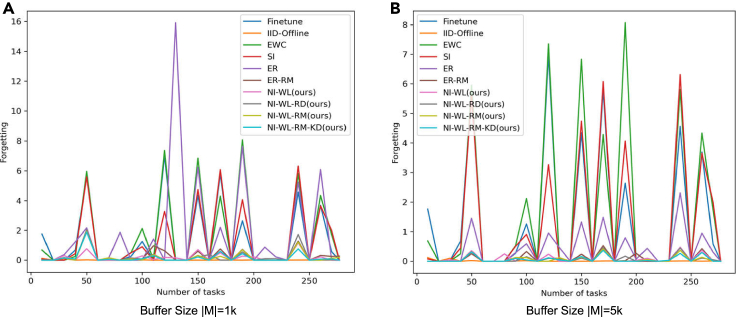
The forgetting metric on test set without random rotation so far measured by the end of each task (object) (A) 1K buffer size is used for replay related methods. (B) 5K buffer size is used for replay related methods.

**Figure 7:**
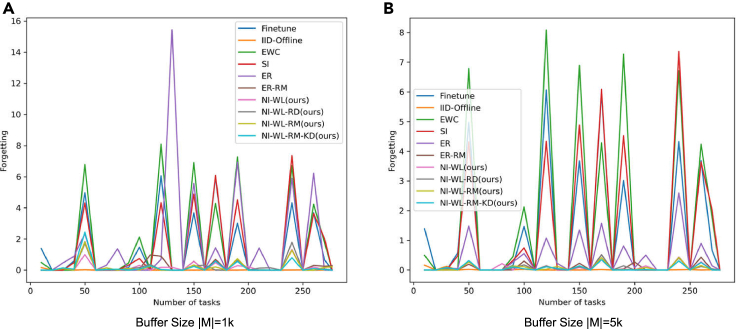
The forgetting metric on test set with random rotation so far measured by the end of each task (object) (A) 1K buffer size is used for replay related methods. (B) 5K buffer size is used for replay related methods.

In Figures 4 and 5, we show how the loss on test set so far evolve along with seeing more tasks, i.e., seeing more objects. Figure 4 is for the version of which test set is without random rotation, while Figure 5 is for that with random rotation. The evolution processes of test forgetting along with training are shown in Figure 6 (without random rotation) and Figure 7 (with random rotation). The lower and smoother the loss is, the better the corresponding CL method is. So is the forgetting. From left to right in Figures 4, 5, 6, and 7, memory size changes with 1K–5K. As shown in Figures 4, 5, 6, and 7, the navie fine-tune has high loss (also high forgetting) and oscillates up and down with a large attitude, indicating catastrophic forgetting occurs. EWC is even worse than Fineturn under 5K buffer size, due to the saturation-prone property of regularization methods in the long steam. SI is better than Fineturn, but is still with high loss, high forgetting, and large oscillation. ER has high loss, high forgetting, and is with large oscillation when the buffer size is small, such as 1K. With the increasing of buffer size, ER performs well gradually. ER is with low loss, low forgetting, and small oscillation when big buffer size is used, such as with 5K memory buffer. Thanks to the diversity of the replayed samples, ER-RM performs well under different buffer size. By contrast, the variants of our proposed method perform better with lower loss, lower forgetting, and smaller oscillation. NI-WL-RM-KD achieves best results, which is very close to the IId-Offline, even with only 1K memory buffer. The visualized tendencies of Figures 4, 5, 6, and 7for alternatives with and without random rotation are similar, indicating that the proposed method is robust to random rotation.

##### Mean average loss and average forgetting

The quantitative results of Figures 4, 5, 6, and 7 are summarized in Table 2, which is provided as the mAL and average forgetting F. Firstly, on test set without random rotation, compared to EWC, SI, ER, and ER-RM, our proposed NI-WL-RM-KD with small buffer size 1K shows ∼39.6%, ∼35.4%, ∼53.9%, and ∼3.5% relative reduction in mAL, ∼90.1%, ∼87.4%, ∼90.8%, and ∼40.7% reduction in forgetting, respectively. This indicates that the proposed NI-WL-RM-KD is able to work under small memory cost. Under other buffer size conditions, four variants of our proposed method, including NI-WL, NI-WL-RD, NI-WL-RM, and NI-WL-RM-KD, consistently outperform other CL methods with a large margin in terms of mAL and forgetting both. Second, a similar trend is shown both on test set without rotation and with rotation, which indicates the robustness of the proposed methods to random rotation. Further, compared to IID-Offline, all CL methods are with more 16% in mean average losses indicating gap still exists.

**Table 2:** Mean average loss mAL and average forgetting F of compared methods

Methods	mAL↓ (TeWoR)^a^	F↓ (TeWoR)	mAL↓ (TeWR)^a^	F↓ (TeWR)
Fine-tune	7.889	1.368	7.924	1.284
IID-Offline	3.470	—b	3.520	—
EWC	8.199	1.712	8.209	1.754
SI	7.657	1.344	7.686	1.382

**BufferSize**|M|=1K				

ER	10.723	1.843	10.554	1.749
ER-RM	5.132	0.285	5.169	0.284
NI-WL^c^	5.630	*0*.*175*	5.543	**0.175**
NI-WL-RD^c^	5.255	0.215	5.243	0.208
NI-WL-RM^c^	*5*.*100*	0.198	*5*.*089*	*0*.*192*
NI-WL-RM-KD^c^	**4.950**	**0.169**	**5.066**	0.194

**BufferSize**|M|=5K				

ER	5.094	0.406	5.147	0.416
ER-RM	5.077	0.096	5.143	0.092
NI-WL	5.144	0.091	5.197	0.076
NI-WL-RD	*4*.*768*	0.071	4.891	0.073
NI-WL-RM	4.855	*0*.*064*	*4*.*887*	*0*.*068*
NI-WL-RM-KD	**4.490**	**0.062**	**4.511**	**0.063**

**BufferSize**|M|=10K				

ER	4.975	0.420	4.980	0.412
ER-RM	4.948	0.082	5.035	0.091
NI-WL	4.996	0.082	4.988	0.079
NI-WL-RD	4.810	**0.058**	4.853	**0.066**
NI-WL-RM	*4*.*558*	*0*.*068*	*4*.*574*	**0.066**
NI-WL-RM-KD	**4.029**	0.069	**4.045**	*0*.*071*

Bold underline, italic underline, and underline font highlights the first place, second place, and third place with same BufferSize, respectively.

a TeWoR is short for test set without rotation, TeWR is short for test set with rotation.

b “—” means not applicable.

c NI-WL, NI-WL-RD, NI-WL-RM, and NI-WL-RM-KD are four variants of our proposed method.

##### Loss on combined test set of finally trained model

In Table 3, we provide the average loss of finally trained models for all compared methods. The losses are calculated on combined test set or combined training set of all experiences. There are four losses for each method, namely, loss on test set without rotation (Loss-test-w/o-rot), loss on test set with rotation (Loss-test-w/-rot), loss on training set without rotation (Loss-train-w/o-rot), and loss on training set without rotation (Loss-train-w/-rot). As demonstrated in Table 3, four losses of Fineturn are quite high and are all above 5.25. As expected lower bound, losses of IID-Offline are low and below 3.6. Consistent with evolution of test loss along with training in Figures 4 and 5, EWC has high losses around 6.9 which are all larger than those of Fineturn, losses of SI are around 4.9 and are slightly lower than Fineturn’s. Losses of ER appear similar tendency with Figures 4 and 5, is high under small buffer size, while is low under big buffer size. ER-RM performs better than ER due to its diversity of replay samples. For the variants of our proposed method, ER-WL-RD gets the most significant drop of loss under 1K buffer size, from 11.267 of ER to 4.356. This indicates the weakly learned plus diverse sapling enhanced the representative and diversity of replay samples. The full-armed version of our proposed method, i.e., ER-WL-RM-KD, gets the most top places.

**Table 3:** Loss L on combined set (test set or training set) of finally trained models for compared methods

Methods	L↓ (TeWoR)^a^	L↓ (TeWR)^a^	L↓ (TrWoR)^a^	L↓ (TrWR)^a^
Fine-tune	5.299	5.257	5.298	5.256
IID-Offline	3.519	3.571	3.521	3.572
EWC	6.986	7.071	6.984	7.074
SI	4.899	4.938	4.897	4.934

**BufferSize**|M|=1K				

ER	11.267	10.982	11.283	10.991
ER-RM	5.186	5.195	5.187	5.192
NI-WL^b^	5.172	5.061	5.172	5.060
NI-WL-RD^b^	**4.356**	**4.391**	**4.358**	**4.391**
NI-WL-RM^b^	4.766	4.830	4.769	4.831
NI-WL-RM-KD^b^	*4*.*631*	*4*.*782*	*4*.*632*	*4*.*776*

**BufferSize**|M|=5K				

ER	4.182	**4.188**	4.182	**4.188**
ER-RM	4.596	4.759	4.599	4.760
NI-WL	4.740	4.790	4.737	4.792
NI-WL-RD	*4*.*172*	4.386	*4*.*170*	4.388
NI-WL-RM	4.400	4.319	4.402	4.320
NI-WL-RM-KD	**4.162**	*4*.*200*	**4.168**	*4*.*197*

**BufferSize**|M|=10K				

ER	**3.754**	**3.799**	**3.755**	**3.800**
ER-RM	4.616	4.707	4.614	4.704
NI-WL	4.607	4.513	4.609	4.514
NI-WL-RD	4.542	4.510	4.540	4.508
NI-WL-RM	4.182	4.182	4.180	4.180
NI-WL-RM-KD	*4*.*028*	*4*.*045*	*4*.*029*	*4*.*047*

Bold underline, italic underline, and underline font highlights the first place, second place, and third place with same BufferSize, respectively.

a TeWoR is short for test set without rotation, TeWR is short for test set with rotation, while TrWoR is short for training set without rotation, and TrWR is short for training set with rotation.

b NI-WL, NI-WL-RD, NI-WL-RM, and NI-WL-RM-KD are four variants of our proposed method.

#### Results in simulation

To illustrate the effectiveness of the proposed approach on continually generating anthropomorphic grasps with high quality, we conduct simulated experiments in the physics-based simulator MuJoCo.^22^ 58 objects from YCB (yale-cmu-berkeley) dataset^23^ (seen) and 48 objects from EGAD!^24^ (unseen) are used. For each object, the completed 3D point cloud is taken as the input of the trained grasp model $M_{g}$, and $M_{g}$ generates 20 grasps randomly. In the simulator, we perform grasp with generated grasp configuration for all used objects and calculate the metrics, i.e., SR, Penetration Depth, and Penetration Volume between the hand mesh and the target object. The steps in the physical simulation process are described in Section "steps for the simulated experiments" of STAR Methods.

**Figure 8:**
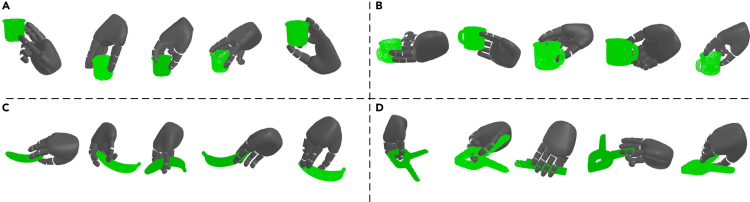
Qualitative grasps generated by NI-WL-RM-KD with 5K memory buffer on 4 objects from YCB object set (A) Grasps for object ycb 065-f cups scaled. (B) Grasps for object ycb 025. (C) Grasps for object ycb 011 banana scaled. (D) Grasps for object ycb 052 extra large clamp scaled.

**Figure 9:**
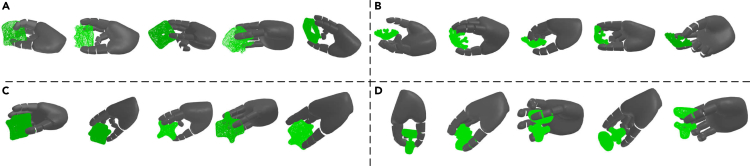
Qualitative grasps generated by NI-WL-RM-KD with 5K memory buffer on 4 objects from EGAD! object set (A) Grasps for object G5. (B) Grasps for object G6. (C) Grasps for object E1. (D) Grasps for object E4.

In Table 4 and Table 5, the compared results on grasping seen objects from YCB and unseen objects from EGAD! in the simulation are provided respectively. As shown in Table 4, most top places with respect to SR on grasping objects from YCB are achieved by our proposed approach. At the same time, the variants of our proposed method, NI-WL, NI-WL-RD, NI-WL-RM, and NI-WL-RM-KD, are consistently with lower Penetration. Table 5 shows a similar tendency also. Overall, the proposed approach outperforms other alternatives on grasping object in simulation with higher SR and lower penetration including depth and volume. Moreover, it is observed that ER-WL for different buffer size, ER-WL-RD, ER-WL-RM, and ER-WL-RM-KD under 5K and 10K buffer size, outperform IID-Offline for unseen EGAD! object dataset, perhaps due to the bias from the dominant objects in IID-Offline. Qualitative results shown in Figures 8 and 9 demonstrate that our proposed method is able to generate diverse reasonable grasps.

**Table 4:** Compared results on grasping seen objects from YCB in simulation

Methods	P-Depth^a^$\left(cm\right)↓$	P-Volume^a^$\left(cm^{3}\right)↓$	Success Rate (%)↑
Fine-tune	0.824	9.142	32.8
IID-Offline	0.642	7.273	62.0
EWC	0.714	8.530	23.3
SI	0.733	8.663	33.6

**BufferSize**|M|=1K			

ER	1.262	23.204	41.4
ER-RM	0.895	11.928	49.3
NI-WL^b^	**0.516**	**6.115**	**54.7**
NI-WL-RD^b^	0.755	10.073	45.3
NI-WL-RM^b^	*0*.*731*	9.963	*50*.*6*
NI-WL-RM-KD^b^	0.818	*6*.*413*	47.1

**BufferSize**|M|=5K			

ER	0.668	8.021	53.0
ER-RM	**0.477**	*5*.*211*	55.2
NI-WL	*0*.*496*	5.868	**55.5**
NI-WL-RD	0.533	6.587	54.7
NI-WL-RM	0.531	5.908	54.5
NI-WL-RM-KD	0.613	**4.400**	*55*.*4*

**BufferSize**|M|=10K			

ER	0.608	6.340	*55*.*4*
ER-RM	*0*.*455*	4.703	**55.9**
NI-WL	0.512	5.720	52.7
NI-WL-RD	**0.438**	*4*.*458*	54.6
NI-WL-RM	0.482	5.201	53.1
NI-WL-RM-KD	0.661	**4.355**	51.3

Bold underline, italic underline, and underline font highlights the first place, second place, and third place with same BufferSize, respectively.

a P-Depth is short for Penetration Depth, P-Volume is short for Penetration Volume.

b NI-WL, NI-WL-RD, NI-WL-RM, and NI-WL-RM-KD are four variants of our proposed method.

**Table 5:** Compared results on grasping unseen objects from EGAD! in simulation

Methods	P-Depth^a^$\left(cm\right)↓$	P-Volume^a^$\left(cm^{3}\right)↓$	Success Rate (%)↑
Fine-tune	0.668	5.526	45.2
IID-Offline	0.562	6.951	73.0
EWC	0.446	3.341	32.2
SI	0.535	2.654	44.6

**BufferSize**|M|=1K			

ER	1.123	17.455	51.9
ER-RM	0.964	20.867	55.5
NI-WL^b^	**0.458**	*6*.*874*	**81.7**
NI-WL-RD^b^	*0*.*760*	13.237	65.3
NI-WL-RM^b^	0.805	16.537	*69*.*4*
NI-WL-RM-KD^b^	0.763	**5.822**	69.0

**BufferSize**|M|=5K			

ER	0.677	*5*.*654*	65.4
ER-RM	**0.448**	6.593	**78.5**
NI-WL	*0*.*486*	7.390	76.9
NI-WL-RD	0.513	8.590	*77*.*9*
NI-WL-RM	0.645	9.317	73.4
NI-WL-RM-KD	0.536	**3.686**	76.9

**BufferSize**|M|=10K			

ER	0.474	**3.092**	74.0
ER-RM	0.473	6.326	**76.6**
NI-WL	0.488	6.942	76.0
NI-WL-RD	**0.407**	5.310	74.1
NI-WL-RM	*0*.*432*	5.912	73.8
NI-WL-RM-KD	0.636	*5*.*181*	*76*.*5*

Bold underline, italic underline, and underline font highlights the first place, second place, and third place with same BufferSize, respectively.

a P-Depth is short for Penetration Depth, P-Volume is short for Penetration Volume.

b NI-WL, NI-WL-RD, NI-WL-RM, and NI-WL-RM-KD are four variants of our proposed method.

## Discussion

In this work, the problem of continual anthropomorphic grasping is considered. In particular, a NICAG approach is developed, which incorporates several discovered biological neural mechanisms supporting continual lifelong learning and consists of a CL framework of anthropomorphic grasping and a neuro-inspired CL algorithm. The experiments carried out on dataset and in simulation provide encouraging results, showing that this approach achieves better CL capability with lower average loss and forgetting, but also gets higher SR for grasping, with reference to some CL metrics and grasp quality metrics. The proposed system offers an approach for endowing anthropomorphic robotic hands with the ability to learn to grasp different objects continually and incrementally over time, and has great potential to make a profound impact on robots in households and factories.

Starting from this work, some future directions are worthwhile considering. Firstly, dealing with more task settings of continual anthropomorphic grasping, one example is CL of anthropomorphic grasping for different purposes (e.g., tool use, hand over, pick, and place), or with different hands. Secondly, due to reinforcement learning could be utilized to reduce repeated failures, integrating supervised learning and reinforcement learning into the continual anthropomorphic grasping framework is also a good direction. Thirdly, developing composite continual anthropomorphic grasping systems that incorporate more biological mechanisms of lifelong learning and human grasping is of great significance. Some biological mechanisms to be incorporated include neuromodulation,^25^ context-dependent perception and gating,^26^ and cognition outside the brain.^27^ Finally, the development of realistic test environments that specifically address CL capabilities of anthropomorphic grasping is another crucial factor for the advancement of continual grasping technology and need to be explored further.

### Limitations of the study

Our approach only utilizes simulated data to improve the grasp performance and it is validated on dataset and in a simulator. Our demonstration here can be the first step toward a combination of neuro-inspired CL and anthropomorphic grasping. In the future, it not only needs to validate our approach on real robotics but also needs to find more sophisticated and effective methodologies that enable performance improvement using the data collected from both real task executions and simulators.

## STAR★Methods

### Key resources table

**Table:** 

REAGENT or RESOURCE	SOURCE	IDENTIFIER
**Deposited data**		

YCB dataset	YCBBenchmarks	https://www.ycbbenchmarks.com/object-set/
EGAD!	Dougsm	https://dougsm.github.io/egad/

**Software and algorithms**		

PyTorch	The Linux Foundation https://github.com/pytorch/pytorch	RRID:SCR_018536
Avalanche	ContinualAI	https://github.com/ContinualAI/avalanche
MuJoCo	DeepMind	https://github.com/deepmind/mujoco
Original code	This paper	https://github.com/WanyiLi/NICAG

### Resource availability

#### Lead contact

Further information and any related requests should be directed to and will be fulfilled by the lead contact, Peng Wang (peng wang@ia.ac.cn).

#### Materials availability

This study did not generate new unique reagents.

### Method details

#### Anthropomorphic robotic hand

The used anthropomorphic robotic hand is DLR/HIT Hand II.^28^ It has five modular fingers with four joint and three active degrees of freedoms. We use a vector with 20 component $\theta \in R^{20}$ to denote the joint configuration of the hand. The wrist pose p of DLR/HIT Hand II is given in special Euclidean group SE(3), consisting of the translation $t=\left[t_{x},t_{y},t_{z}\right]$ and orientation quaternion $q=\left[q_{w},q_{x},q_{y},q_{z}\right]$.

#### The used grasp model

For grasp model $M_{g}$ of which state expects to be updated by continual learning algorithm $A_{CL}$, we adopt the variational grasp generator which is the core module of DVGG^10^ as a case study. We ignore the two auxiliary steps including object point completion and iterative grasp refinement for clarity.

#### Description of compared methods

We compare the proposed approach with four typical continual learning approaches including elastic weight consolidation (EWC),^18^ synaptic intelligence (SI),^19^ experience replay (ER)^20^ and rainbow memory (ER-RM),^21^ and two baselines, namely, Finetune and IId-Offline. EWC^18^ and SI^19^ overcome forgetting with importance-based regularization. ER^20^ is a simple but effective replay-based approach, which applies reservoir sampling^29^ for memory update and random sampling for memory retrieval. Rainbow memory (ER-RM)^21^ enhances diversity of samples in a representative memory via a novel memory management strategy based on uncertainty and data augmentation. Diverse samples in memory are replayed to overcome forgetting. Finetune incrementally finetunes the model without employing any continual learning strategy. Finetune can not overcome catastrophic forgetting and is considered as the naive baseline. IId-Offline uses all the samples in the dataset in an offline manner to train the model, and is regarded as the oracle baseline.

#### Dataset

To evaluate our proposed framework and methods for continual learning of anthropomorphic grasping, we construct a sequential anthropomorphic grasping dataset based on Wei et al. . The used anthropomorphic robotic hand is DLR/HIT Hand II. There are more than one million grasp samples on 300 objects. We firstly remove those objects with few effective grasps, as a result, 278 objects are preserved. And then, we build a continual grasp learning setting, the data in the setting is modeled as an ordered sequence composed of 278 non-iid learning experiences, a learning experience is a set of grasp samples from an individual object, as shown in Figure 10. The complete 3D point cloud of each object is taken as observation P. For each experience, we split the grasp samples into training set, validation set and test set at the ratio of 6:2:2. Training set and validation set in the sequence are used to train the grasping models continually, while the test set is used to test trained models.

#### Steps for the simulated experiments

The simulated experiments are conducted in the physics-based simulator MuJoCo.^22^ There are four steps in the physical simulation process: 1) Fix the object stationary and initialize the robotic hand with a pre-grasp state, then the hand approaches the object and executes grasping with the generated grasp parameters including hand wrist pose and angles of hand joints until a stable state of the simulator reaches. 2) Then the gravity is present, fingers keep the grasping force till a stable simulator state reaches or the object falls from the hand. 3) By shaking the hand, the unstable grasps are filtered, and grasps that keep the object in hand are preserved as successful ones. 4) Calculate the metrics including Success Rate (SR), Penetration Depth and Penetration Volume, as mentioned in experimental results.

## Data Availability

• Data reported in this paper will be shared by the lead contact upon request. • The code is deposited at the GitHub repository, https://github.com/WanyiLi/NICAG, and is publicly available as of the date of publication. A link to code has been included in the key resources table. • Any additional information required to reanalyze the data reported in this paper is available from the lead contact upon request.
